# Alignment of port policy to the context of the Physical Internet

**DOI:** 10.1080/03088839.2022.2147594

**Published:** 2023-03-13

**Authors:** Patrick B.M. Fahim, Gerjan Mientjes, Jafar Rezaei, Arjan van Binsbergen, Benoit Montreuil, Lorant Tavasszy

**Affiliations:** a Transport & Logistics Group, Faculty of Technology, Policy & Management, Delft University of Technology, Delft, The Netherlands; b Transport & Planning Group, Faculty of Civil Engineering & Geosciences, Delft University of Technology, Delft, The Netherlands; c Physical Internet Center, H. Milton Stewart School of Industrial & Systems Engineering, Georgia Institute of Technology, Atlanta, GA, USA

**Keywords:** Physical Internet (PI), freight transport, ports, policy, scenarios, multi-criteria decision analysis (MCDA)

## Abstract

The Physical Internet (PI) is a paradigm-changing and technology-driven vision, which is expected to significantly impact the development of the freight transport and logistics (FTL) system of today. However, the development of the FTL system towards the PI creates much uncertainty for its current stakeholders. Ports are one of those stakeholders that are expected to be profoundly affected by these developments. However, research that focuses on port policy, under the uncertain developments towards the PI, is still lacking. By providing port authorities with insights and recommendations on robust policy areas, we address this void in literature. We conduct a scenario analysis in combination with multi-criteria decision analysis (MCDA) to determine the importance of port performance indicators and policy areas in different scenarios. The most significant, uncertain, and orthogonal factors for the development of the PI are technological development and institutional development. We find that for a proper alignment with the PI vision, in three out of four scenarios, ports should prioritize the implementation of digital solutions and standards, as opposed to an infrastructure focused policy.

## 1. Introduction

Freight transport and logistics (FTL) contribute around 15% to the world’s GDP and account for over 10% of a finished product’s costs on average (Mervis 2014). Simultaneously, among others, by transportation marking its presence with over 30% of the global carbon emissions (IEA 2019), today’s FTL system is often considered as non-sustainable from an economic, environmental, and societal perspective (Montreuil, Meller, and Ballot 2013). Additionally, as demonstrated by regular disruptions with resulting shock-effects on international trade and manufacturing, the system suffers from vulnerabilities and lack of resilience. Besides being fundamental components of the FTL system, ports function as critical facilitators of international trade, through which they contribute to the economic development of countries and regions. With 80% of the total merchandise trade being transported over sea, annual global maritime trade volumes have surpassed 10 billion tons (Arvis et al. 2018). Whereas ports’ economic value creation and complexity increase over time (Lee and Lam 2016), they need to continuously adapt to their external environment due to changing economic and trading patterns, new technologies, legislation, and port governance systems (Nijdam & Van der Horst, 2017).

The Physical Internet (PI), a paradigm-changing and technology-driven vision, is expected to impact these current economic and trading patterns, technologies, legislation, and governance systems. In his seminal paper, Montreuil (2011, 71) positioned the PI as an all-encompassing vision ‘for the future of how physical objects are transported, handled, stored, supplied, realized, and used across the world’. By analogy with the digital internet (DI) (Van Luik et al. 2020), the PI proposes physical shipments to be encapsulated into multi-level modular containers, which autonomously find their way through an open hyperconnected network of logistics networks. More recently, Montreuil (2020, 2) defined the PI as a ‘hyperconnected global logistics system enabling seamless open asset sharing and flow consolidation through standardized encapsulation, modularization, protocols and interfaces’, where it aims to seamlessly connect physical, informational and financial flows. The development towards the PI is expected to have a profound impact on the functioning of today’s FTL system. Although it has been recognized as a promising vision by both academia and industry, the development is uncertain for many current stakeholders in the FTL system and requires collaborative research by academia, industry, and government.

Ports are expected to be significantly affected by the developments towards the PI (Fahim et al. 2021a). Port authorities (PAs), as central actors in ports, aim to synchronize the interests and actions of public stakeholders with private stakeholders, including their own strategic intents (Van der Lugt, de Langen, and Hagdorn 2017). Since ports and their infrastructures are extremely asset heavy with high investment costs and needs, a thorough understanding of the way the FTL system develops towards the PI is crucial to determine a correct allocation of investment resources and sustainable long-term policymaking for ports. In this paper, we define port policy as a set of strategic activities and measures that enable long-term development, planning, and learning to enhance overall port performance and attractiveness to its users.

This long-term development is fraught with uncertainty. Fahim et al. (2021a) constructed an evolutionary PI port development framework with potential future development paths. In the context of policy-making and decision-making under uncertainty, a combination of scenario analysis and multi-criteria decision analysis (MCDA) is expected to yield meaningful results. Earlier, Fahim et al. (2022) analyzed intelligent agents’ port performance evaluation and selection preferences in the context of the PI. However, the study on which port policy fits best to the diverse possible contexts of the PI has so far been unexplored. The research question to be answered in this paper is as follows:

### 1.1. ‘What are suitable policy areas for port authorities in the development towards the Physical Internet?’

By answering this question, we aim to provide PAs with insights and recommendations on robust policy areas towards the PI, where our expectation is that the optimal selection of policies will depend on the prevailing scenario towards the PI. We aim to contribute to literature on (1) maritime port policy and management in the technology-driven and paradigm-changing vision of the PI, and (2) policy selection in uncertain environments, through a combination of scenario analysis and MCDA.

The remainder of the paper is organized as follows. Section 2 provides a review of the relevant streams of literature for our research in the domains of the PI, future ports, and port policy. Section 3 presents the methodological approach. In Section 4, the construction of the contextual PI scenarios is described, the PI port performance indicators (PPIs) are presented, and the potential PI port policy areas are proposed. In Section 5, the aggregated results are presented and discussed with implications and recommendations for PAs. Section 6 ends the paper by means of a conclusion and recommendations for future research.

## 2. Literature review

### 2.1. PI fundamentals

By positioning the PI as addressing the Global Logistics Sustainability Grand Challenge, the PI is receiving increasing attention from academia, industry, and governmental institutions. In addition to the earlier provided definition, Montreuil (2020) defined eight Building Blocks for the PI: (1) unified set of standard modular logistics containers; (2) containerized logistics equipment and technology; (3) standard logistics protocols; (4) certified open logistics facilities and ways; (5) global logistics monitoring system; (6) open logistics decisional and transactional platforms; (7) smart data-driven analytics; and (8) certified open logistics service providers. For a review of the wider PI literature, we refer to Treiblmaier et al. (2020); here we highlight only the elements that directly relate to maritime ports.

Synchromodality, i.e. the synchronization between operations of different transport modes, is a fundamental element of the PI (Lemmens, Gijsbrechts, and Boute 2019). Decisions about switching between transport modes and routes are made real-time in response to demand variations, and resource and network availabilities. Another core element of the PI lies in the concept of collaboration between stakeholders by sharing physical and digital assets. Simmer et al. (2017) stress the need for collaboration and its governance in the PI, while admitting to barriers stemming from mainly trust between stakeholders. Ballot, Gobet, and Montreuil (2012a) showed results of potential network cost savings, ranging from 4% to 26%, along with a potential threefold reduction in harmful emissions. From a PI container perspective, Montreuil, Ballot, and Tremblay (2016) suggest that they should be smart, connected, eco-friendly, modular, and easy to handle, store, transport, designed following global standards and exploitable across multiple modes of transportation.

From a PI hub perspective, Ballot, Montreuil, and Thivierge (2012b), Montreuil et al. (2012), Montreuil et al. (2012), and Montreuil, McGinnis, and Buckley (2021) addressed conceptual designs of intermodal hubs, road-based transit centers, road-based cross-docking hubs, and parcel logistics hubs, respectively, while Karakostas (2019) used queuing theory to calculate properties such as throughputs, processing times, and traffic size. Focusing on maritime ports, Fahim et al. (2021a) constructed an evolutionary PI port development framework to position changes over time, whereas Fahim et al. (2022) analyzed the changes that can be expected in intelligent agents’ port performance evaluation and selection preferences. Fahim et al. (2021b) addressed general managerial implications of the evolution of the PI for port and maritime practitioners.

### 2.2. Future ports

In comparison with today, future ports will increasingly need to address the capability of real-time information sharing among its stakeholders, high-end technology-driven IT solutions, sustainability, physical and digital port connectivity, and value-added services (VAS) (Lee and Lam 2016; Montreuil et al. 2018; Chu et al. 2018; Ha, Yang, and Lam 2019; Fahim et al. 2021a). The evolutionary framework in Fahim et al. (2021a) describes the evolution from today’s ports into globally hyper-connected ports in a fully functioning global PI, the PI Port Framework (PIPF). The PIPF shows that the main aspects, which influence the development of current ports into PI ports, can be captured in governance, operational, and digital dimensions. Also in the PI, Fahim et al. (2022) identified costs, level of service, network interconnectivity, geographical location, reliability, and information systems (ISs) as most important indicators for port performance. Montreuil et al. (2018), however, stress that, to create a fully functioning PI, standardization of interfaces and protocols is a prerequisite. Ports need to develop digital capabilities which provide intelligence, automation, and visibility, i.e. tracking-and-tracing (T&T), not only on container level but also on individual shipment level (Fahim et al. 2021c).

In achieving hyperconnectivity, interconnected and interoperable ISs (of the different stakeholders), together with data and information (exchange) platforms, such as Port Community Systems (PCSs), have a crucial role and can accelerate the transition of ports towards the PI (Iida et al. 2019; Caldeirinha, Nabais, and Pinto 2022). PCSs can be described as neutral and open digital platforms, which enable secure exchange of information between both public and private stakeholders. PCSs aim to improve the competitiveness of port communities by automating, optimizing, and managing port and logistics processes through a single submission of data and connecting supply chains (IPCSA 2018). Delenclos, Rasmussen, and Riedl (2018) argue that, to become a next-generation port of the future, ports should aim for the implementation of the same technology-driven innovations that are disrupting other industries: connected ISs and platforms; cloud-based services; sensors and other Internet of Things (IoT) technologies; augmented reality; intelligent decision-making systems; blockchain; and big data analytics applications. Although up-front capital investments are high, port automation results in operational cost savings, while also contributing to performance enhancements and safety gains. Successfully automated ports proved that operational costs could drop between 25% and 55%, and productivity could rise between 10% and 35% (Chu et al. 2018).

### 2.3. Port policy

From a policy perspective, Lam and Notteboom (2014) provide pricing, monitoring, market access control, and environmental standard regulation as four main categories for port policy, while Hou and Geerlings (2016) identify modal shift, technological means, and spatial measures as port policy options. Furthermore, whereas Kang and Kim (2017) refer to technologies, monitoring and upgrading, process and quality improvement, active participation, and communication and cooperation as main dimensions of port policy practices, Aregall, Bergqvist, and Monios (2018) identified technology, dedicated infrastructure, monitoring program, engine regulations, regulatory instruments, intermodal service development, port dues and subsidy funds, certification, knowledge improvement, and concessions as main port policy measures. Bjerkan and Seter (2019) distinguish between concession agreements, collaboration, management of environment and energy, modal split, monitoring, port dues, and other managerial policies as main categories within port policy and management. A distinction can be made between investments in existing capabilities (e.g. hinterland accessibility, throughout capacity) and future capabilities (e.g. IoT technologies, intelligent decision-making systems) of ports. Being the organizations that are responsible for the management and development of a competitive, sustainable, and safe port environment, PAs need to make strategic decisions on investments within the geographical boundaries of the port area, but also beyond, in the fore- and hinterland (Notteboom and Lam 2018). Simultaneously, PAs need to strategically position themselves among and towards both private and public port stakeholders (Van der Lugt, de Langen, and Hagdorn 2017) in an environment that is experiencing a process of (horizontal) collaboration and (vertical) integration.

### 2.4. Positioning of the study

Although over the past decade the number of publications, research areas and applied methods within the PI have been growing fast, the topic of maritime ports is underrepresented in the PI literature. Research that focuses on the way ports could design policy under the uncertain development of the FTL system towards the PI is lacking. By investigating the vision of the PI in the context of port policy, we aim to contribute with this topic to the stream of PI literature as well as to the port policy and management literature.

## 3. Methodology

### 3.1. Overall approach

The approach comprises a combination of *scenario analysis* and *MCDA*. We use scenario analysis to identify alternative and plausible futures in the PI context. We define an effective policy as one that maximizes a port’s attractiveness for its potential users, by influencing port performance in the relevant dimension. Hence, policy effectiveness will depend on

- The relative importance of each port performance indicator to its users, and
- The relative impact of each port policy area on each indicator.

Both these factors are highly context-dependent. Depending on the degree to which the PI has been realized, users may emphasize different performance criteria (e.g. attach more importance to costs, or service quality) and it may therefore result in different dimensions of port performance becoming important. For example, when the PI is far advanced, investments in physical infrastructure may be less meaningful than in digital infrastructure. Our approach involves measuring I and II above separately and combining them into one policy effectiveness indicator. We measure relative importance of the *port performance indicators in each scenario* and the *impact of the policy areas on the port performance indicators in each scenario* using an MCDA method named Best-Worst Method (BWM) (Rezaei 2015) .

Next, using a weighted sum method, we can determine the *overall effectiveness of the policy areas in each scenario*, as follows.

(1) $$E_{\mathrm{ps}}=\overset{n}{\underset{i=1}{\sum }}w_{\mathrm{is}}I_{\mathrm{pi}}$$

where

$E_{\mathrm{ps}}$ is the effectiveness of policy area *p* in scenario *s*;

$w_{\mathrm{is}}$ is the relative importance (weight) of performance indicator *i* for overall port attractiveness in scenario *s;*

$I_{\mathrm{pi}}$ is the impact of policy area *p* on indicator *i*.

**Figure 1.**
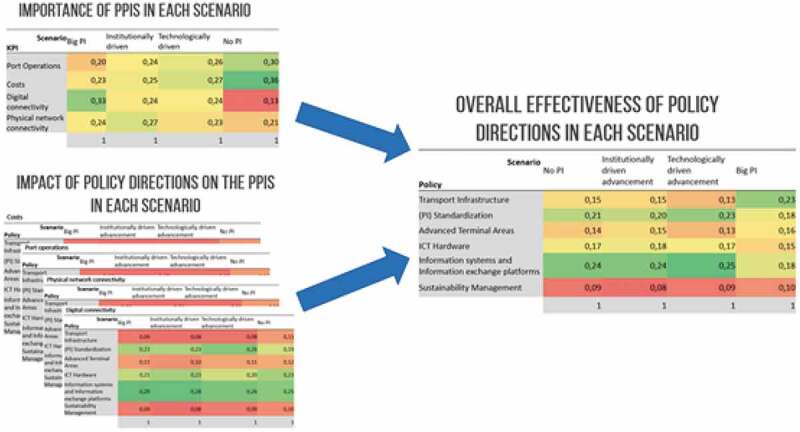
Evaluating the effectiveness of the six policy areas for four PPIs in four scenarios.

Figure 1 illustrates how effectiveness of the six policy areas is assessed for the four PPIs under the four scenarios. We elaborate on the detailed contents of these tables further on in the paper.

**Figure 2.**
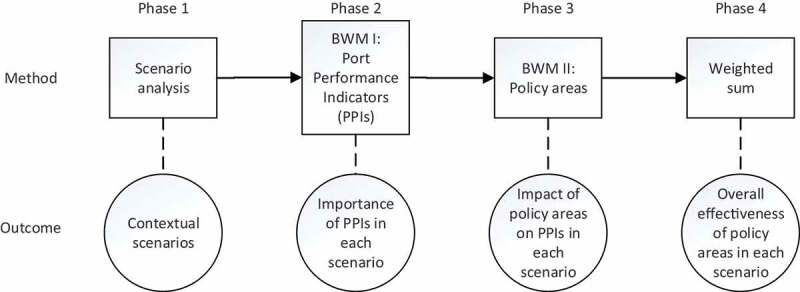
Research process.

Figure 2 depicts the overall research process with respective consecutive phases.

In the next sections, we subsequently discuss the scenario analysis and MCDA implementation approach.

### 3.2. Scenario analysis

In order to account for the uncertainty around the development of the PI, we formulate explorative scenarios using *scenario logic* (Enserink et al. 2010). The first step is to identify *contextual factors*. These are the variables that influence the development, performance, and outcome of a particular system, while lying outside the influence of the *problem owner*. We derive these contextual factors from a literature review. In the second step, the contextual factors are clustered into *driving forces*. These can be considered as the main underlying clusters of variables with the highest levels of *uncertainty* and *impact* that influence the system. In the third step, by means of attributing opposing (positive/+ and negative/-) development directions to the driving forces, the scenario logic is constructed, and scenarios are obtained. Using this approach, the number of obtained scenarios is equal to ‘2 to the power of the number of selected driving forces’. By balancing practicability and the provision of meaningful results, we aim for a set of between three and eight scenarios. This is in line with previously conducted transport scenario studies (e.g. Port of Rotterdam 2019; Inkinen, Helminen, and Saarikoski 2021).

### 3.3. Multi-criteria decision analysis (MCDA) implementation

MCDA is a sub-field of operations research where multiple decision alternatives are analyzed with respect to multiple (often conflicting) decision criteria. Among several MCDA methods, we choose the BWM. It is a data-efficient method and has proven to produce consistent and reliable results (Rezaei 2015). Through the initial selection of the best and worst criteria, to which the other criteria are compared, BWM is structured, easily executable, and time-efficient. By means of its pairwise comparisons, the BWM also helps decision-makers to gain additional valuable insights. Moreover, through the use of only integers, fundamental distance problems which might occur with the use of fractions in pairwise comparisons can be prevented (Rezaei 2015). Finally, the use of two opposite references (best and worst) mitigates a potential anchoring bias of the respondent (Rezaei 2020).

Our empirical research approach is built around the MCDA method and comprises four steps: (1) establishing criteria, (2) surveying experts, (3) determining weights, and (4) aggregating the results.

We used BWM for finding the weights of the *port performance indicators in each scenario* and the *impact of the policy areas on the port performance indicators in each scenario*. To avoid confusion, we call the first implementation of BWM as BWM I and the second implementation as BWM II.

**Step 1** establishes a set of criteria. For BWM I, the PPIs represent the set of criteria. These are identified by means of literature review. For BWM II, the policy areas represent the set of criteria. To identify these policy areas, in addition to a literature review, we conduct a series of 14 semi-structured digital ‘face-to-face’ expert interviews. We use a semi-structured interview approach to be able to give the interviewees some direction, while allowing them to freely express their opinions and complement the discussions. We select the 14 experts on the basis of their experience with ports and/or PI, from academia and industry. Appendix A provides a list of the 14 expert interviewees with respective functions and affiliations.

**Step 2** obtains experts’ preferences as input data for the BWM. For the evaluation of the importance of the PPIs in the different scenarios in BWM I, a survey among 14 experts is conducted. For the evaluation of the effectiveness of the policy areas on the PPIs in the different scenarios in BWM II, a survey among 21 experts is conducted. In both cases, these experts are selected based on their academic experience with ports and/or PI, their (scientific) contributions to ports and/or PI, and/or industry experience. Appendix B provides the lists of experts with respective functions and affiliations that participated in the surveys of BWM I and BWM II.

**Step 3** determines the relative priorities/weights by means of the BWM. Since, in both BWM implementations, we are dealing with the preferences of a group of experts, we employ the Bayesian BWM. The Bayesian BWM is a probabilistic variant of BWM, which is specifically designed to obtain the relative priorities/weights of criteria for a group of DMs (Mohammadi and Rezaei 2020a). Next to obtaining the relative priorities/weights, an additional valuable feature of the Bayesian BWM is that it provides ranking schemes. These ranking schemes are called credal rankings and are able to measure the degree to which a group of DMs prefers one criterion over another by means of a confidence level (Mohammadi and Rezaei 2020b). The higher the confidence level, the more certain the group shows to be about a relationship between the two criteria. Appendix C provides a more elaborate explanation of the Bayesian BWM.

## 4. Results

In this section, we first describe the four scenarios that we obtained by scenario analysis. Secondly, we introduce the performance indicators and present their relative importance in the different scenarios. Thirdly, we present the identified policies that ports could use in their policy design, as well as their effectiveness on performance indicators in the different scenarios. Fourthly, we present the aggregated results in terms of the overall effectiveness of the policy areas in each of the scenarios.

### 4.1. Scenarios

For the scenario analysis, 27 external factors were identified that influence the global FTL system. Since we aim to incorporate all facets of the all-encompassing vision of the PI, and since both the technological and institutional components are crucial in the development towards the PI, we cluster the external factors into two driving forces: (1) *Technological development* and (2) *Institutional development*. We chose these two driving forces due to their high impact on the FTL system and high uncertainty in its developments, while simultaneously being able to provide a high aggregate level and all-encompassing analysis. Appendix D provides an overview of the 27 external factors with the corresponding driving forces.

**Figure 3.**
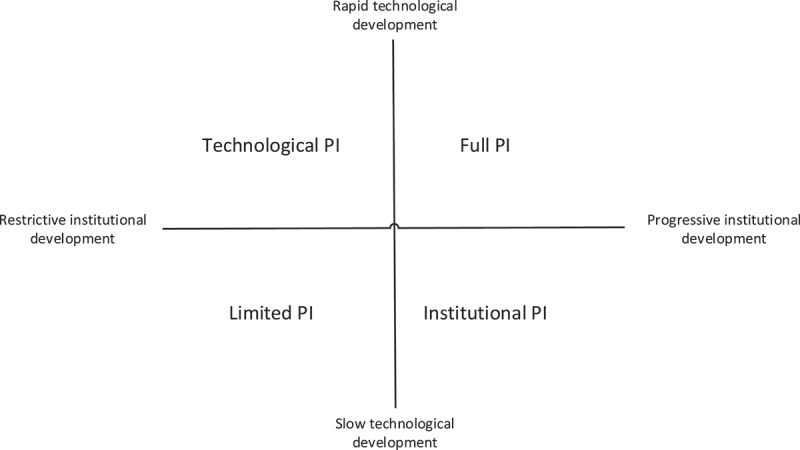
Scenario logic for PI ports.

For both driving forces, a positive and a negative future outcome is envisioned. In terms of technological development, a positive future outcome constitutes a *fast technological development*, whereas a negative future outcome constitutes a *slow technological development*. Similarly, in terms of institutional development, a positive future outcome constitutes a *progressive institutional development*, whereas a negative future outcome constitutes a *restrictive institutional development*. These potential positive and negative future outcomes are opposites on the two axes, which represent the driving forces of a scenario logic (see Figure 3), as prescribed by Enserink et al. (2010). By combining these potential positive and negative future outcomes of the two driving forces, four different scenarios towards the PI are created by means of the quadrants of the scenario logic.

### 4.2. Scenario 1: Limited PI

In the *Limited PI* scenario, due to slow technological developments, (implemented) solutions and applications in the fields of IoT, big data analytics and AI, and blockchain remain limited. Additionally, developments in cloud and edge computing (power) also lack behind, because of which both the autonomous real-time decision-making capabilities of intelligent agents, and the overall (hyper)connectivity between FTL stakeholders and entities do not come into fruition, as necessary for a well-functioning PI. Furthermore, from an institutional perspective, limited development in (PI) standards with a reluctance to collaborate and share resources by FTL stakeholders, partially, due to a lack in collaborative business models, and cohesive legal and regulatory frameworks, hinder the PI from moving forward. In this particular PI port scenario, the status quo of the FTL system remains and the PI will still be in its infancy by 2030.

### 4.3. Scenario 2: Institutional PI

In the *Institutional* PI scenario, the PI is driven by progressive institutional developments. Through the development and implementation of (PI) standards alongside the development and adoption of collaborative business models, and cohesive legal and regulatory frameworks, FTL stakeholders are willingly collaborating and sharing their physical and digital resources. Additionally, the modular PI containers are widely adopted in the FTL system. However, since technological developments are lagging behind with limitations, notably in the fields of IoT, big data analytics, AI, and blockchain, the PI operations are not being optimized. Additionally, developments in cloud and edge computing (power) also lag behind, because of which both the autonomous real-time decision-making capabilities of intelligent agents and the overall (hyper)connectivity between FTL stakeholders and entities do not materialize. Hence, although the FTL stakeholders are collaborating, from a technological perspective, this scenario presents a similar FTL as we know today. A fully functioning PI is not yet to be expected by 2030.

### 4.4. Scenario 3: Technological PI

In the *Technological PI* scenario, technological development is fast and provides opportunities to implement the PI. Due to the wide adoption of technologies and applications related to IoT, big data analytics and AI, and blockchain, operations in the FTL system are being optimized. Additionally, developments in cloud and edge computing (power) are rapidly advancing, enabling autonomous real-time decision-making by intelligent agents and the overall (hyper)connectivity between FTL stakeholders and entities. However, due to lacking collaborative business models alongside legal and regulatory restrictions, FTL stakeholders still prove to be reluctant towards collaborating and sharing resources. Also, the development of (PI) standards is lagging behind. Hence, although technological innovations are being rapidly developed and implemented, through which the FTL system becomes smart and operations become more optimized and efficient, stakeholders are not fully hyperconnected and collaborating due to a lack in the adoption of (PI) standards and the willingness to share resources. In this scenario, a fully functioning PI is not yet to be expected by 2030.

### 4.5. Scenario 4: Full PI

In the *Full PI* scenario, rapid technological development is paired with progressive institutional development. The rapid technological development provides opportunities to implement the PI on a global scale. Implemented technologies and applications related to IoT, big data analytics and AI, and blockchain allow operations in the FTL system to be optimized. Additionally, developments in cloud and edge computing (power) are rapidly advancing, enabling intelligent agents to autonomously make real-time decision and the envisioned hyperconnectivity between FTL stakeholders and entities. Furthermore, through the development and implementation of (PI) standards alongside the development and adoption of collaborative business models, and cohesive legal and regulatory frameworks, FTL stakeholders are willingly collaborating and sharing their resources. Also, because of both technological and institutional advancement, modular PI containers and interoperable digital information platforms are widely adopted in the FTL system. In this particular scenario, a fully functioning PI is expected by 2030, through which the FTL system becomes more sustainable from an economic, environmental, and societal perspective.

### 4.6. Port performance indicators (PPIs)

The analysis of port performance evaluation by its users has important implications for a port’s policy formulation and investment decisions. Whereas traditional port users, i.e. decision-makers, are represented by shippers, shipping lines, and logistics service providers, the routing protocol of the PI will require intelligent agents, i.e. intelligent containers and vessels, to support or replace current port users as decision-makers. Although port performance evaluation in a contemporary context has been abundantly investigated (e.g. Arvis et al. 2018; Ha, Yang, and Lam 2019), only Fahim et al. (2022) addressed this topic in the advanced context of the PI. Hence, the indicators, which we use in this research to evaluate intelligent agents’ port performance preferences in the different PI port scenarios, are inspired by Fahim et al. (2022). Table 1 tabulates these PPIs with respective descriptions.

**Table 1.** Port performance indicators with respective descriptions.

Port Performance Indicator (PPI)	Description
A. Port Operations (PO)	The overall quality and efficiency of operations regarding container and vessel handling within the port boundaries. This includes factors such as speed, capacity, reliability, agility, flexibility, responsiveness, safety, security, and sustainability.
B. Costs	The costs from the perspective of the port users. These costs include transshipment costs and seaport duties.
C. Digital Connectivity (DC)	The degree to which a port is digitally connected with its own community stakeholders and with other stakeholders of the FTL chain, in both fore- and hinterland.
D. Physical Network Connectivity (PNC)	The degree to which a port is physically connected with its fore- and hinterland. A higher degree of (intermodal) PNC leads to an increased degree in reliability, agility, flexibility, and responsiveness of the overall FTL chains in which a port takes part.

After having obtained the preferences of the group of 14 experts by means of a survey, we employed the Bayesian BWM to compute the relative importance of the indicators in the different scenarios as well as the respective credal rankings. Table 2 presents the relative importance of the performance indicators in the different scenarios.

**Table 2.** Importance of port performance indicators in the different scenarios.

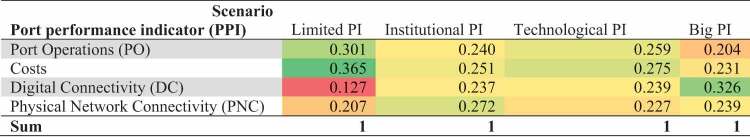

*Costs* and *Port Operations (PO)* are perceived as most important in the Limited PI scenario, which is in line with contemporary port performance evaluation and selection literature. Although the range of values in the importance of the PPIs in an Institutional PI scenario are relatively small, *Physical Network Connectivity (PNC)* and Costs are perceived as most important. This could be interpreted as the two PPIs that are least dependent on the technological development having the highest importance. However, at the same time, although the range of values in the importance of the PPIs also in a Technological PI scenario are relatively small, Costs and PO are perceived as most important. This could indicate that technology is mostly seen as a means for operational productivity and efficiency gains, while simultaneously decreasing costs of operations. *Digital Connectivity (DC)* and PNC are perceived as the most important PPIs in the Full PI scenario, which indicates that the PI is still mostly perceived as a digital innovation with achieving (hyper)connectivity in the FTL system as its main function. Between scenarios, the largest discrepancies can be found in the importance of DC between Full PI and Limited PI (0.326–0.127 = 0.199), and in the importance of Costs between the Limited PI and Full PI (0.365–0.231 = 0.134).

An additional observation is the discrepancy in the range of values (between the most and least important) of the PPIs in the different scenarios: 0.238 (0.365–0.127) in the Limited PI scenario; 0.035 (0.272–0.237) in the Institutional PI scenario; 0.048 (0.275–0.227) in the Technological PI scenario; and 0.122 (0.326–0.204) in the Full PI scenario. We can conclude that a larger range can be found in the more ‘extreme’ scenarios, i.e. Full PI and Limited PI, while smaller ranges can be found in the ‘intermediate’ scenarios, i.e. Institutional PI and Technological PI. Implying that, in the intermediate scenarios, the experts’ preferences in PPIs are more balanced, while, in the more extreme scenarios, the experts have a more distinguished preference.

**Figure 4.**
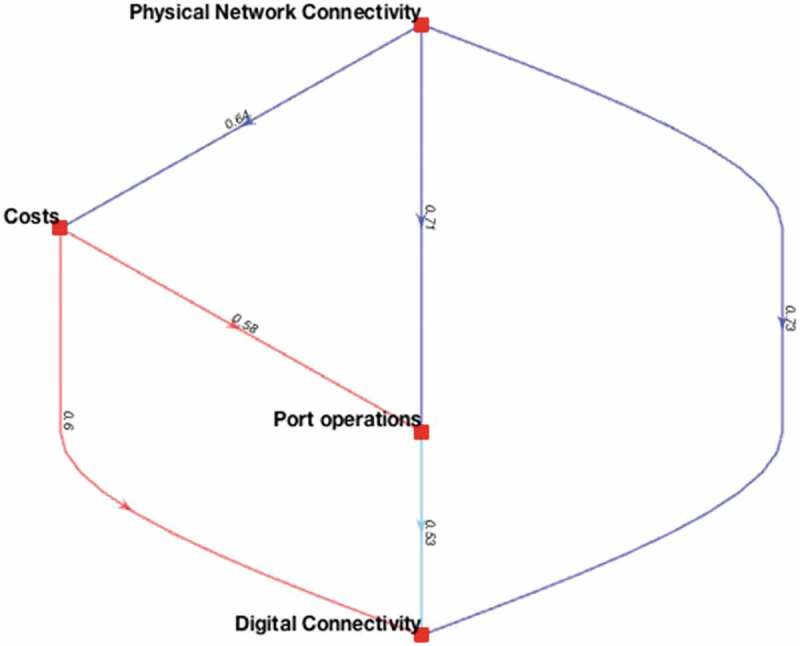
Weighted directed graph with respective credal rankings of the *Institutional PI* scenario.

The credal rankings are visualized in a weighted directed graph, where the nodes represent the importance, and each link $s\overset{v}{\Rightarrow }s^{\prime }$ indicates that indicator s is more important than indicator $s^{\prime }$ with confidence v. Figure 4, for instance, visualizes the weighted directed graph with respective credal rankings of the Institutional PI scenario. Although the guideline is that a confidence level of 0.50 can be used as a threshold value (Mohammadi and Rezaei 2020a), values of 0.53, 0.58, 0.60, and 0.64 in Figure 4 indicate that there is some dissension between the experts’ opinions about those particular relationships. The credal rankings of the Technological PI scenario show similar results, while most of the credal rankings of the Limited PI and Full PI scenarios are in full or almost full confidence levels, which is in line with the observation of the experts having a distinguished preference in importance of port performance indicators in the more ‘extreme’ scenarios. Hence, the conclusion can be drawn that the importance differences of the PPIs in these two scenarios are determined with full or almost full confidence by the group of experts.

### 4.7. Policies

Ports could implement several measures and invest in policies to maintain and improve their attractiveness for port users in the uncertain development towards the PI. To identify policy areas that ports could use in their policy design and strategy formulation, we conducted a literature review and a series of 14 semi-structured expert interviews. Although there are various ways to categorize policies (e.g. Lam and Notteboom 2014; Hou and Geerlings 2016; Kang and Kim 2017; Aregall, Bergqvist, and Monios 2018; Bjerkan and Seter 2019), in this paper, we define six areas that bear particular relevance for ports developing towards the PI. The defined policy areas with respective descriptions are tabulated in Table 3.

**Table 3.** Policy areas with respective descriptions.

Policy area	Description
A: Transport Infrastructure	In improving a port’s attractiveness, a PA could invest in the *transport infrastructure* to enhance the (multi-modal) accessibility of the port, both by land and sea, and increase its capacity (Montreuil et al. 2018). This includes investments, such as the enlargement of the rail shunting yard capacity and the deepening of the waterside access channel to ease draft restrictions so that larger vessels can berth but also beyond the physical port boundaries in terms of developing the hinterland infrastructure, inland- and dry terminals, extended gates, (integrated) rail, offshore and Hyperloop terminals (DP World 2020), and airports. This policy area could contribute to achieving synchromodality.
B: (PI) Standardization	This policy area includes the development of standards, required for, for example, interoperable information systems (ISs), the digitalization of the Bill-of-Lading and customs declarations, nautical standards, physical and digital interfaces, protocols, synchromodality, and modular (PI) containers. PAs could contribute to setting these standards by leading its coordination with other organizations. Once particular standards are chosen and adopted, PAs could further stimulate their implementation and adoption by means of incentives and rules in concession agreements, access regulation, and pricing strategies (Lam and Notteboom 2014; Van der Lugt, de Langen, and Hagdorn 2017; Aregall, Bergqvist, and Monios 2018; Notteboom and Lam 2018).
C: Advanced Terminal Areas	For ports to be capable of conducting smart, real-time dynamic decisions on the container consolidation and synchromodal operations (Montreuil et al. 2018), PAs could invest in the development of dedicated *advanced terminal areas*. Within these, the automated, and later autonomous, (re)positioning and crossdocking of the PI containers could take place (Fahim et al. 2021a; Fahim et al. 2021c).
D: ICT Hardware	Advanced *ICT Hardware* in ports will be necessary to achieve the desired level of connectivity, visibility, and synchormodality in the PI (Fahim et al. 2021b). Additionally, sensors and wireless communication technologies, enable fast- and fact-based exchange of information, which allows ports to become more agile, dynamic, and responsive (Montreuil et al. 2018). The technological readiness of edge- and cloud computing plays a critical in the effectiveness of these decentralized systems (Wang and Sarkis 2021).
E: Information Systems and Platforms	For the enablement of hyperconnectivity and synchromodality to facilitate seamless informational and financial flows, interoperable digital platforms are a prerequisite. PAs are required to integrate their own ISs and stimulate the integration of ISs throughout the supply chain. The PA could improve the smart functionalities of its ISs by applying AI, IoT, and big data analytics (Hasan et al. 2020; Ahmad et al. 2021). Additionally, the PA could play a facilitating role by developing a neutral digital information platform, i.e. PCS, to provide “single version of the truth” informational services. Furthermore, these ISs and platforms could be connected with the fore- and hinterland to digitally integrate ports within a global PI.
F: Sustainability Management	*Sustainability management* is the policy area that aims to address the negative externalities. PAs can take measures to comply with, among others, environmental regulation and working conditions, while also developing monitoring systems to maintain safety, air quality, water quality, and control nuisances (Lam and Notteboom 2014; Di Vaio, Varriale, and Alvino 2018). Additionally, emergent digitalization and information technologies are expected to bring opportunities that positively impact the environmental supply chain sustainability (Sarkis, Kouhizadeh, and Zhu 2021). Furthermore, PAs can encourage other port stakeholders to prioritize sustainability, for example, by incentives and rules in the concessions, access regulation, and pricing strategies.

After having obtained the preferences of a group of 21 experts by means of a survey, we employed the Bayesian BWM to compute the relative impact of the policy areas on each of the PPIs in the different scenarios as well as the respective credal rankings. Table 4 presents the impact of the policy areas on the PPIs in the different scenarios. However, since all the credal rankings were far above the threshold value of 0.5, which indicates that the difference of the impact of the policy areas in the different scenarios are determined by the group of experts with confidence, we decided not to show the weighted directed graphs.

**Table 4.** Impact of policy areas on the PPIs in the different scenarios.

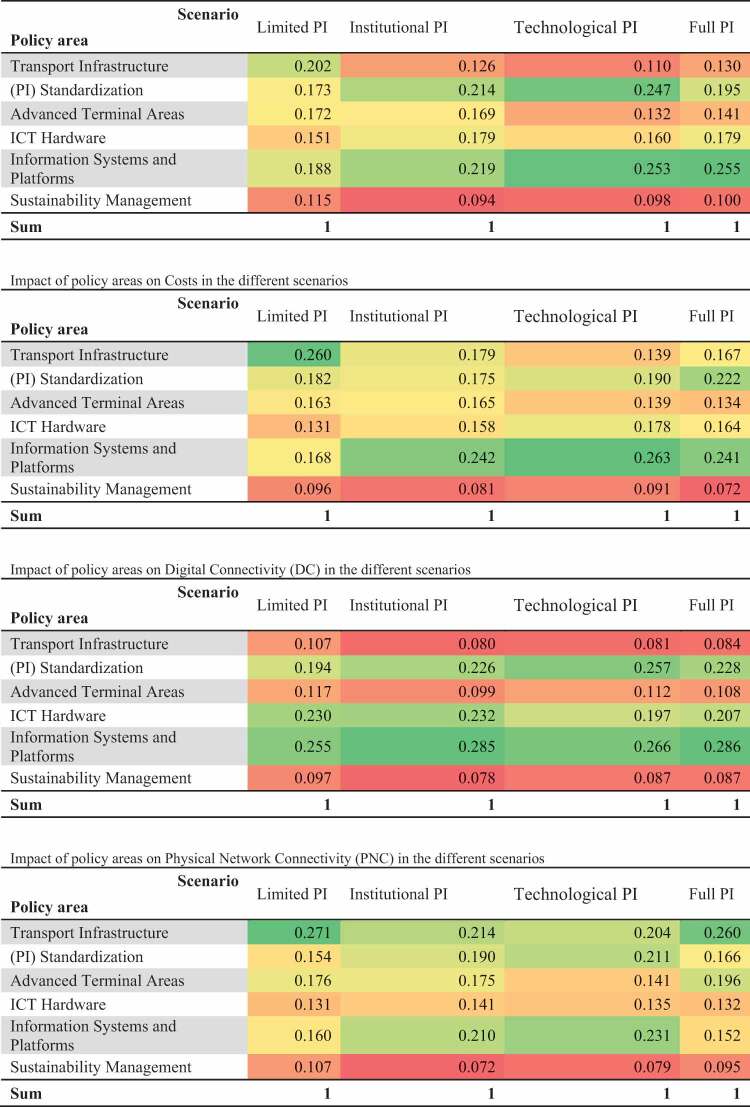

It becomes evident that Information Systems and Platforms and (PI) Standardization are the most impactful policies on PO and Costs in all scenarios, except in the Limited PI scenario where Transport Infrastructure is considered most impactful. Regarding DC, we can observe that, in addition to (PI) Standardization and Information Systems and Platforms, ICT Hardware is considered a significantly impactful policy area in all scenarios. With respect to PNC, Table 4 indicates that Transport Infrastructure is the most impactful policy area in all scenarios, except in the Technological PI scenario where Information Systems and Platforms is considered the most impactful policy area. Another clear observation is that Sustainability Management is the least impactful policy area on all PPIs in all scenarios.

### 4.8. Aggregated results

The aggregated results, i.e. *the overall effectiveness of the policy areas in each scenario*, are obtained by combining the *importance of the PPIs in each scenario* with the *impact of the policy areas on the PPIs in each scenario* (see Eq. 1). These aggregated results are tabulated in Table 5.

**Table 5.** Overall effectiveness of policy areas in the different scenarios.

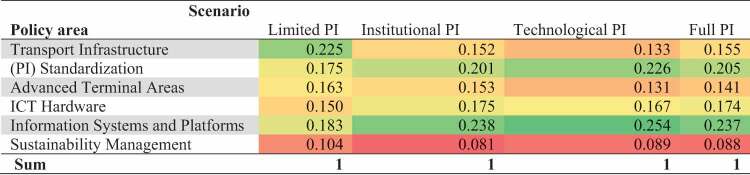

We observe that Information Systems and Platforms is the most effective policy, followed by (PI) Standardization. This counts for the Full PI scenario and the two intermediate scenarios, Institutional PI and Technological PI. In the Limited PI scenario, Transport Infrastructure is considered most effective. Advanced Terminal Areas and ICT Hardware are considered similarly effective in all scenarios, whereas Sustainability Management is considered least effective in all scenarios. With respect to Sustainability Management, it must be taken into account, however, that the effectiveness is evaluated against the PPIs from a user perspective.

## 5. Discussion

Reflecting on the importance of the port performance indicators in the different scenarios, we can observe that there is a highest-importance movement from the highest importance of Costs and Port Operations in the Limited PI scenario to Digital Connectivity and Physical Network Connectivity in the Full PI scenario. This movement is in line with the contemporary port performance evaluation and selection literature (e.g. Ha, Yang, and Lam 2019), which prioritizes high-quality logistics services against lowest costs, as does the PI literature (e.g. Montreuil et al. 2018).

Reflecting on the overall effectiveness of the policy areas in the different scenarios, we can observe that, when moving further towards the Full PI scenario, Information System and Platforms becomes the most effective policy area, which can also be considered to be in line with literature on future ports (e.g. Chu et al. 2018; Delenclos, Rasmussen, and Riedl 2018; Ha, Yang, and Lam 2019) and ports in the PI (e.g. Fahim et al. 2021b, 2022). Additionally, this observation can be considered to be in line with the development of a sixth generation of ports, succeeding the fifth generation of ports as described by Lee and Lam (2016). Additionally, the standardization is one of the essential cornerstones of the PI (Montreuil et al. 2018).

The low effectiveness of Sustainability Management can be attributed to the fact that as long as port users do not value sustainability and the environment as they value other port performance indicators in practice. Hence, although sustainability within freight transport and logistics and green ports has received much attention and importance in literature (e.g. Ashrafi et al. 2020), the question remains whether port authorities will be willing to focus and significantly invest in the Sustainability Management policy area.

When considering the implications of the effectiveness of the policy areas for ports altogether, however, it must be kept in mind that ports are still very dissimilar (Delenclos, Rasmussen, and Riedl 2018). More detailed and specific measures could follow from more specific case studies.

A fundamental challenge of increasingly complex multi-stakeholder networks to overcome is the matter of trust, transparency, and interoperability. Blockchain technology might have the potential to alleviate these concerns (Hasan et al. 2020). Neutral Information Systems and Platforms, such as PCSs, could play a facilitating role here. Port authorities can play a role here by acting as pioneers with the goal to create network effects, convincing other stakeholders to follow suit.

Another major technical challenge is the development and adoption of intelligent infrastructure, such as sensors, wireless communication technologies, and data centers. Part of the challenge here lies in the cloud and edge computing power capabilities, safety, and security, i.e. cybersecurity, and scale of implementation (Wang and Sarkis 2021). Port authorities, again, can play a role here by acting as pioneers with the goal to create network effects, convincing other stakeholders to follow suit.

Lastly, Standardization goes hand in hand with cooperative business models and the enabling legal and regulatory frameworks (Treiblmaier et al. 2020). PAs could lead collaborative efforts with organizations such as the World Trade Organization (WTO), International Maritime Organization (IMO), International PCS Association (IPCSA), Digital Container Shipping Association (DCSA), International Taskforce Port Call Optimization (ITPCO) but also with other freight transport and logistics stakeholders.

Finally, an important limitation of the research concerns the small group of respondents. Although they have been selected based on their knowledge of the constituent domains and have created a logical and internally consistent picture, different respondents may have produced different results. The validity of this picture outside of the current context of European ports remains to be assessed.

## 6. Conclusions and future research

The main objective of this paper is to provide ports with insights and recommendations on robust policy areas towards the PI, given its highly uncertain development. Therefore, the main research question that was formulated in the beginning of this paper is *What are suitable policy areas for port authorities in the development towards the Physical Internet?*

To answer this question, we have proposed a quantitative approach to clarify the effect of uncertainties in the environment of ports on the required policies. The method is shown to discriminate clearly between alternative policies, indicating which policies are robust in which scenarios. To our understanding, this approach is new in the scientific literature in the domain of maritime ports. Future port policy studies can build on this approach.

Our main findings include the following. The most significant, uncertain, and orthogonal factors to consider in mapping potential futures for the development of the freight transport and logistics system into the PI are *technological development* and *institutional development*. Additionally, we show that the pace and approach of the realization of the PI are very relevant for policymaking, as scenarios have shown to have different emphasis on policies. Also, we found that, moving into the PI, the connectivity indicators, *Digital Connectivity* and *Physical Network Connectivity*, become more important, while *Costs* and *Port Operations* become less important for port users. Furthermore, we identified the following policy areas for ports: *(1) Transport Infrastructure, (2)* (PI) *Standardization, (3) Advanced Terminal Areas, (4) ICT Hardware, (5) Information Systems & Platforms*, and *(6) Sustainability Management*. Here, we found that, moving into the PI, Information Systems & Platforms followed by (PI) Standardization are considered most effective.

The implications of the research are that ports should prioritize the development and implementation of digital (IT) solutions and systems that increase productivity and decrease costs of operations, while simultaneously increase supply chain interconnectivity and visibility. In addition, the research shows that standardization will be a necessary means to achieve seamless flow of goods and information between networks and stakeholders in the future context of the PI. The main implication for port managers is that they have to closely monitor the pace of realization of the PI and make sure that they shift their strategy in a timely and suitable manner, from the current policy focus on infrastructure development to a dominant digitalization and standards creating focus in their policymaking.

As avenues for future research, we propose the following. As our current policy areas are still formulated at a rather high level; we recommend to further operationalize them and assess them in more detail, to support their implementation. Additionally, adding a timeline to these measures could help policymakers to create a (dynamic) policy roadmap with respective concrete measures. Furthermore, it is recommended to conduct a cost–benefit analysis to assess the cost-effectiveness of the policy areas. Also, other quantitative analyses could be conducted to gain more insights into the impact of the policy areas, i.e. modelling what the effects of the policy areas are on quantitative indicators, such as container throughput, emissions, and revenue, in the different scenarios. The latter could be done using quantitative freight models and simulations. Lastly, since we mainly used European interviewees and survey respondents, we recommend research around the general applicability of our findings in other major parts of the world.

## Appendix A: List of expert interviewees

**Table A1.** Expert interviewees.

Function	Affiliation
Full Professor of Freight and Logistics	Delft University of Technology, The Netherlands
Full Professor of Global Supply Chains and Ports	Erasmus University Rotterdam, The Netherlands
Full Professor of Supply Chain and Logistics	Georgia Institute of Technology, USA
Innovation Manager	Groningen Seaports, The Netherlands
Full Professor of Maritime Logistics	Kedge Business School, France
Full Professor of Logistics	Kühne Logistics University, Germany
Full Professor of Industrial Engineering and Supply Chain	Mines Paris Tech, France
Innovation Manager	Port of Amsterdam, The Netherlands
Innovation Manager	Port of Algeciras, Spain
Strategist	Port of Rotterdam, The Netherlands
Innovation Director	Port of Valencia, Spain
Full Professor of Transport, Logistics and Ports	University of Antwerp, Belgium
Assistant Professor of Operations and Supply Chain	University of Groningen, The Netherlands
Full Professor of Industrial Engineering	University of Groningen, The Netherlands

## Appendix B: List of survey respondents

**Table B1.** Survey respondents BWM I.

Function	Affiliation
Associate Professor of Operations and Supply Chain Management	Delft University of Technology, The Netherlands
Full Professor of Freight and Logistics	Delft University of Technology, The Netherlands
Full Professor of Multi-Machine Operations and Logistics	Delft University of Technology, The Netherlands
Researcher of Maritime Ports	Delft University of Technology, The Netherlands
Innovation Manager	Groningen Seaports, The Netherlands
Associate Professor of Maritime Logistics	Kedge Business School, France
Full Professor of Logistics	Kühne Logistics University, Germany
Consultant	Maritime shipping consulting company, The Netherlands
Innovation Manager	Port of Algeciras, Spain
Project Manager	Port of Hamburg, Germany
Strategist	Port of Rotterdam, The Netherlands
Full Professor of Transport, Logistics and Ports	University of Antwerp, Belgium
Assistant Professor of Operations and Supply Chain	University of Groningen, The Netherlands
Full Professor of Industrial Engineering	University of Groningen, The Netherlands

**Table B2.** Survey respondents BWM II.

Function	Affiliation
Associate Professor of Operations and Supply Chain Management	Delft University of Technology, The Netherlands
Full Professor of freight and logistics	Delft University of Technology, The Netherlands
Full Professor of Multi-Machine Operations and Logistics	Delft University of Technology, The Netherlands
Researcher of Maritime Ports	Delft University of Technology, The Netherlands
Full Professor of Global Supply Chains and Ports	Erasmus University Rotterdam, The Netherlands
Full Professor of Ports and Transport Economics	Erasmus University Rotterdam, The Netherlands
Innovation Manager	Groningen Seaports, The Netherlands
Consultant	Independent maritime shipping consultant, Denmark
Associate Professor of Maritime Logistics	Kedge Business School, France
Full Professor of Logistics	Kühne Logistics University, Germany
Consultant	Maritime shipping consulting company, The Netherlands
Innovation Manager	Port of Algeciras, Spain
Innovation Manager	Port of Barcelona, Spain
Project Manager	Port of Hamburg, Germany
Strategist	Port of Rotterdam, The Netherlands
Head of Strategy & Analytics	Port of Rotterdam, The Netherlands
Innovation Director	Port of Valencia, Spain
Full Professor of Transport, Logistics and Ports	University of Antwerp, Belgium
Assistant Professor of Operations and Supply Chain	University of Groningen, The Netherlands
Full Professor of Industrial Engineering	University of Groningen, The Netherlands
Full Professor of Operations Research and Management	University of Groningen, The Netherlands

## Appendix C: Explanation of the Bayesian BWM

Assume that *K* experts evaluate *n* criteria *C *= {*c_1_*, …, *c_n_*}. To apply the Bayesian BWM, we should follow these four steps (Mohammadi and Rezaei 2020a):

**Step a**: Expert *k* first selects the best ($c_{B}^{k}$) and the worst ($c_{W}^{k}$) criteria from *C*.

Every expert selects the best and the worst criteria from the set of earlier defined criteria. The best is considered the most important, whereas the worst criterion is considered the least important.

**Step b**: Expert *k* makes the pairwise comparison between the best criterion ($c_{B}^{k}$) and the other criteria.

Every expert expresses his/her preferences of the best criterion to the other criteria on a 9-point scale. Table C shows the scale numbers with respective linguistic variables of the 9-point scale. The pairwise comparison of expert *k* results in the ‘Best-to-Others’ vector $A_{B}^{k}$ as

(2) $$A_{B}^{k}=(a_{B1}^{k},a_{B2}^{k},\ldots ,a_{Bn}^{k}),k=1,2,\ldots ,K,$$

where $a_{Bj}^{k}$ represents the preference of the best criterion ($c_{B}^{k}$) over criterion $c_{j}\in C$ for expert k.

**Table C1.** Nine-point scale with linguistic variables

Scale number	Linguistic variable
1	Equally important
3	Moderately more important
5	Strongly more important
7	Very strongly more important
9	Extremely more important
2, 4, 6, 8	Intermediate values

**Step c**: Expert *k* makes the pairwise comparison between the worst criterion ($c_{W}^{k}$) and the other criteria. Here, every expert expresses his/her preferences of the other criteria over the worst criterion, again, on a 9-point scale. The pairwise comparison of expert *k* in step results in the ‘Others-to-Worst’ vector $A_{W}^{k}$ as

(3) $$A_{W}^{k}=(a_{1W}^{k},a_{2W}^{k},\ldots ,a_{nW}^{k}{)}^{T}$$

where $a_{jW}^{k}$ represents the preference of criterion $c_{j}\in C$ over the worst criterion for expert k ($c_{W}^{k}$).

**Step d**: Obtaining the aggregated weights $w^{*}=(w_{1}^{*},w_{2}^{*},\ldots ,w_{n}^{*}$) and the weight for each expert $w^{k},k=1,\ldots ,K$ based on the following probabilistic model:

$A_{B}^{k}|w^{k}$~ *multinomial*(1/*w^k^*), ∀*k* = 1, …, *K*,

$A_{W}^{k}|w^{k}$~ *multinomial*(*w^k^*), ∀*k* = 1, …, *K*,

*w^k^* | *w** ~ Dir(*ɣ* x *w**), ∀*k* = 1, …, *K*,

*ɣ* ~ *gamma*(0.1, 0.1),

*w** ~ *Dir*(1), (4)

where *multinomial* is the multinomial distribution, *Dir* is the Dirichlet distribution and *gamma* (0.1, 0.1) is the gamma distribution with shape parameters of 0.1.

Considering that this model does not have a closed-form solution, Markov-chain Monte Carlo (MCMC) methods such as ‘just another Gibbs sampler’ (JAGS) must be used (Mohammadi and Rezaei 2020a). The useful outcome of the model is the posterior distribution of weights for every single expert and the aggregated *w**. However, the confidence of the superiority cannot be determined by solely comparing the weights. Therefore, in addition to providing the weights for every single expert and the aggregated $w^{*}$, the Bayesian BWM allows us to calibrate the degree to which one criterion is superior to another by means of a credal ranking.

Prior to providing a definition of the credal ranking, the credal ordering, which is the building block for the credal ranking, will be defined:

**Definition 1** (credal ordering). For a pair of criteria $c_{i}$ and $c_{j}$, the credal ordering *O* is defined as

$$O=\left(c_{i},c_{j},R,d\right)$$

where

- R is the relation between criteria $c_{i}$ and $c_{j}$, i.e. <, >, or = ;
- $d\in \left[0,1\right]$ represents the confidences of the relation.

**Definition 2** (credal ranking). For a set of criteria $C=\left(c_{1},c_{2},\ldots ,c_{n}\right)$, the credal ranking is a set of credal orderings which includes all pairs $\left(c_{i},c_{j}\right)$, for all $c_{i},c_{j}\in C$.

The property of being able to assess the confidence of the superiority provides the DMs with more information that can significantly contribute to their decisions. It is considered even more important since we are dealing with group decision-making (Mohammadi and Rezaei 2020a).

Another Bayesian test can now be devised to find the confidence of each credal ordering. The test is predicated on the posterior distribution $w^{*}$. The confidence that $c_{i}$ being superior to $c_{j}$ is computed as

(5) $$P\left(c_{i}>c_{j}\right)=\int I_{\left(w_{i}^{*}>w_{j}^{*}\right)}P\left(w^{*}\right)$$

where $P\left(w^{*}\right)$ is the posterior distribution of $w^{*}$ and I is 1 if the condition in the subscript holds, and 0 otherwise. This integration can be approximated by the samples via the MCMC. Having *Q* samples from the posterior distribution, the confidence can be computed as

$$P\left(c_{i}>c_{j}\right)\frac{1}{Q}\overset{Q}{\underset{q=1}{\sum }}I(w_{i}^{q*}>w_{j}^{q*})$$

(6) $$P\left(c_{j}>c_{i}\right)\frac{1}{Q}\overset{Q}{\underset{q=1}{\sum }}I(w_{j}^{q*}>w_{i}^{q*})$$

where $w^{q*}$ is the $q^{\mathrm{th}}$ sample of $w^{*}$ from the MCMC samples. Thus, for each pair of criteria, the confidence that one is superior over another can be computed. The credal ranking can be changed into a traditional ranking. Then, it is evident that $P\left(c_{i}>c_{j}\right)+P\left(c_{j}>c_{i}\right)=1$. Hence, $c_{i}$ is more important than $c_{j}$ if, and only if, $P\left(c_{i}>c_{j}\right)>0.5$ (Mohammadi and Rezaei 2020b). As a result, the traditional ranking of criteria can be obtained by applying a threshold of 0.5 in the credal ranking.

## Appendix D: Driving forces with external factors

**Table D1.** Driving forces with external factors

Driving force	External factor	Author(s)
Technological development	Internet of Things (IoT)	Delenclos, Rasmussen, and Riedl (2018); Inkinen, Helminen, and Saarikoski (2021); Meindl et al. (2021)
	Artificial intelligence (AI)	Delenclos, Rasmussen, and Riedl (2018); Meindl et al. (2021); Wang and Sarkis (2021)
	Blockchain/distributed ledger technology	Hasan et al. (2020); Ahmad et al. (2021); Wang and Sarkis (2021)
	(Big) Data analytics	Delenclos, Rasmussen, and Riedl (2018); Meindl et al. (2021); Wang and Sarkis (2021)
	Digitalization	Chu et al. (2018); Inkinen, Helminen, and Saarikoski (2021); Wang and Sarkis (2021)
	Digital information platforms	Delenclos, Rasmussen, and Riedl (2018); IPCSA (2018); Fahim et al. (2021b); Wang and Sarkis (2021)
	Hyperloop	DP World (2020); Montreuil (2020)
	3D Printing	Inkinen, Helminen, and Saarikoski (2021); Meindl et al. (2021); Wang and Sarkis (2021)
	Robotics	DB Schenker (2021); Inkinen, Helminen, and Saarikoski (2021); Meindl et al. (2021)
	Drones	DB Schenker (2021); DHL (2021); Inkinen, Helminen, and Saarikoski (2021)
	Sensors	Delenclos, Rasmussen, and Riedl (2018); Inkinen, Helminen, and Saarikoski (2021); Meindl et al. (2021)
	Automation	Chu et al. (2018); Inkinen, Helminen, and Saarikoski (2021); Meindl et al. (2021)
	Cloud/Edge computing	Delenclos, Rasmussen, and Riedl (2018); Meindl et al. (2021); Wang and Sarkis (2021)
	Intelligent systems	Delenclos, Rasmussen, and Riedl (2018); DB Schenker (2021); Inkinen, Helminen, and Saarikoski (2021); Meindl et al. (2021)
	5(/n)G network	Delenclos, Rasmussen, and Riedl (2018); Inkinen, Helminen, and Saarikoski (2021); Wang and Sarkis (2021)
Institutional development	Environmental regulations	Sarkis, Kouhizadeh, and Zhu (2021); DHL (2021); Fahim et al. (2021a); Inkinen, Helminen, and Saarikoski (2021)
	Global network integration	DHL (2021); Fahim et al. (2021a)
	Modularity/Containerization	Montreuil, Ballot, and Tremblay (2016); (2018)); DHL (2021)
	(Cyber)security	DHL (2021); Inkinen, Helminen, and Saarikoski (2021)
	(PI) Standardization	Montreuil et al. (2018); Fahim et al. (2021a); Fahim et al. (2021b); 2021c; 2022); Inkinen, Helminen, and Saarikoski (2021)
	(Collaborative) Business models	Treiblmaier et al. (2020); Inkinen, Helminen, and Saarikoski (2021); Meindl et al. (2021); Wang and Sarkis (2021)
	Labour protection	DHL (2021); Meindl et al. (2021)
	Trade agreements	Fahim et al. (2021a); Inkinen, Helminen, and Saarikoski (2021)
	Antitrust policies	Fahim et al. (2021a)
	Willingness to share (data and assets)	Treiblmaier et al. (2020); DHL (2021); Inkinen, Helminen, and Saarikoski (2021)
	Regulatory frameworks	Treiblmaier et al. (2020)
	Legal frameworks	Treiblmaier et al. (2020); Inkinen, Helminen, and Saarikoski (2021)
